# Transcriptome analysis of early embryonic development in a mouse model of polycystic ovary syndrome

**DOI:** 10.3389/fcell.2025.1554437

**Published:** 2025-05-02

**Authors:** Shan Han, Jiale Lv, Xuedong Sun, Yanqiu Xie, Yuhua Shi

**Affiliations:** ^1^ Guangdong Cardiovascular Institute, Guangdong Provincial People’s Hospital, Guangdong Academy of Medical Sciences, Guangzhou, China; ^2^ Department of Reproductive Medicine, Guangdong Provincial People’s Hospital (Guangdong Academy of Medical Sciences), Southern Medical University, Guangzhou, China; ^3^ Department of Reproductive Medicine, Nanfang Hospital, Southern Medical University, Guangzhou, China

**Keywords:** polycystic ovary syndrome, mouse model, prenatal exposure, pre-implantation embryo, RNA-seq

## Abstract

Polycystic ovary syndrome (PCOS) is a complex disorder that originates during fetal development and significantly impairs female fertility during the reproductive years. Although it is hypothesized that prenatal exposure to elevated androgen levels plays a crucial role in the pathogenesis of PCOS, the precise effects of such exposure on the offspring of individuals with PCOS remain unclear. In this study, we established a mouse model of PCOS by administering dihydrotestosterone (DHT) prenatally. We subsequently evaluated the reproductive phenotype and fertility of the PCOS-like mice, focusing on ovarian function and embryo developmental potential. Smart-seqII RNA sequencing was performed on blastocysts to obtain the RNA expression profile of preimplantation embryos from PCOS mice. These findings indicate that the PCOS model mice exhibit hyperandrogenic symptoms, reduced ovulation rates, and impaired development of oocytes and blastocysts compared to controls. Furthermore, 918 differentially expressed genes were identified in the blastocyst samples, with significant enrichment in pathways related to intracellular energy metabolism, tissue development, glycolipid metabolism, hormone synthesis, and inflammation. This research presents direct evidence that prenatal exposure to hyperandrogenism negatively influences the early embryonic development of offspring and plays a significant role in the later manifestation of polycystic ovary syndrome in adulthood. These findings contribute valuable insights for the early prevention of PCOS.

## Introduction

Polycystic ovary syndrome (PCOS) represents the most prevalent reproductive metabolic disorder among women of reproductive age and is frequently associated with infertility, obesity, cardiovascular diseases, and psychological disorders, such as depression, all of which significantly compromise women’s physical and mental health. The global prevalence of PCOS ranges from 6% to 20%, contingent upon the diagnostic criteria employed. Over the past 3 decades, there has been an increase of more than 29.5% in the global annual incidence rates of PCOS, underscoring its emergence as a significant public health concern (Stener-Victorin et al., 2024; Yang et al., 2022; Shokeir and Abdelshaheed, 2022). Statistical evidence suggests that approximately 80% of individuals with PCOS encounter infertility issues (Ennab and Atiomo, 2023). Moreover, due to factors such as diminished oocyte quality and disrupted hormone levels, individuals with PCOS are at an elevated risk for infertility and pregnancy complications (Escobar-Morreale, 2018). Furthermore, PCOS demonstrates a significant familial and heritable component, with approximately 60%–70% of daughters born to women with PCOS developing the PCOS phenotype during puberty and young adulthood (Gorsic et al., 2017; Gorsic et al., 2019; Dapas et al., 2019). This characteristic substantially threatened the reproductive health of both patients and offspring. Despite being a human-specific condition, the intricate etiology and prolonged duration of polycystic ovary syndrome pose challenges in directly employing patient offspring for investigations into disease prevalence and transgenerational correlations. Consequently, the development of appropriate animal models has emerged as a focal point of research aimed at elucidating the mechanisms underlying the transmission of PCOS to progeny.

It should be noted that erandrogenemia remains the most heritable phenotypic trait (Legro et al., 1998), despite the considerable heterogeneity in clinical manifestations and comorbidities observed among patients with polycystic ovary syndrome. According to the diagnostic criteria established by the Hyperandrogenism and PCOS Association, the presence of hyerandrogenic manifestations is crucial for diagnosing polycystic ovary syndrome (Biesbroek et al., 2018; Azziz et al., 2006). Hyperandrogenemia or its clinical manifestations are observed in over 60% of patients. To the patients with PCOS, hyperandrogenemia is also associated with metabolic disorders. The prevalence of severe obesity has increased to over 25% among individuals with PCOS and hyperandrogenemia (Escobar-Morreale, 2018; Neven et al., 2018; Kataoka et al., 2019), which significantly impact health and lifestyle. A species-specific clinical diagnostic criterion for PCOS in animals is currently lacking. Nonetheless, certain animal models exhibit two or more of the Rotterdam criteria, suggesting PCOS-like characteristics. Therefore, classifying these models as PCOS-like could improve their utility in etiological research. Based on this, animal models of PCOS have been extensively studied across a range of species, including mice (Moore et al., 2013), rats (Hu et al., 2015), sheep (Cardoso and Padmanabhan, 2019), and non-human primates (Abbott et al., 2008). Among these, mice are regarded as the most valuable and extensively researched mammalian models for investigating the intergenerational transmission of PCOS. This is attributed to their rapid growth and reproductive rates, substantial genomic homology with humans, and the advanced feasibility of gene editing and modification techniques. In the existing literature, androgen-induced polycystic ovary syndrome mouse models have been instrumental in elucidating certain phenotypic manifestations observed in PCOS patients (Risal et al., 2019; Bozdag et al., 2016). These models consistently demonstrate hallmark metabolic abnormalities, such as impaired glucose tolerance, insulin resistance, and dyslipidemia. Furthermore, AR knockout (ARKO) mouse models exhibiting complete or partial androgen receptor insufficiency confer protection against hyperandrogenism-induced phenotypic manifestations of PCOS in female mice. These protective effects include the prevention of acyclicity, ovulatory dysfunction, and adipocyte hypertrophy (Caldwell et al., 2015). In light of the predominant hypothesis that hyperandrogenism plays a crucial role in the pathogenesis of polycystic ovary syndrome, mice models of PCOS induced by high doses of androgens, typically utilizing agents such as testosterone and dihydrotestosterone (DHT), have gained widespread acceptance in contemporary research (Stener-Victorin et al., 2020).

Building upon recent findings from animal models, numerous hypotheses have been proposed regarding the pathogenesis of polycystic ovary syndrome. Numerous studies have demonstrated that daughters of women with polycystic ovary syndrome are at a significantly heightened risk of developing the condition themselves (Risal et al., 2019). Female macaques naturally experiencing high androgen levels show PCOS-like traits, possibly starting from mid-gestation (Risal et al., 2019). With over 95% DNA similarity in protein-coding regions, rhesus macaques are closely related to humans, supporting the idea that PCOS may have developmental origins. However, devising safe and ethical experimental methodologies to explore the mechanisms underlying this phenomenon in human subjects presents considerable challenges. In contrast to postnatal models that depend on prolonged high doses of exogenous treatment, the prenatally androgenized (PNA) animal model offers a promising approach for examining the transmission of PCOS to offspring. The existing literature has shown that PNA mice exhibit phenotypic abnormalities, including increased anogenital distance, irregular estrous cycles, increased fat mass, enlarged adipocytes, elevated liver triglyceride levels, and reduced respiratory exchange ratio and energy expenditure, despite being completely protected from adverse environmental exposures postnatally (Liu et al., 2025). These results support that exposure to adverse environmental factors, such as hyperandrogen encountered during early life stages contribute to the onset of PCOS in adulthood, and underscores a distinct pattern of intergenerational and transgenerational inheritance.

Clinical investigations into early embryonic development associated with polycystic ovary syndrome have demonstrated that hyperandrogenemia adversely affects preimplantation embryonic progression. In a prospective cohort study examining two pronuclei (2 PN) embryos derived from hyperandrogenic PCOS patients and control subjects, the impact of PCOS on early embryonic development was evaluated through time-lapse analysis of embryonic kinetics from fertilization to the blastocyst stage. In comparison to the control group, embryos from hyperandrogenic PCOS patients exhibited a significant delay in 2 PN breakdown and progressed slowly from fertilization to the 8-cell stage (Wissing et al., 2014). The detrimental impact of androgen exposure on early embryonic development has been partially corroborated through a limited number of studies utilizing animal models. Immunostaining of cleavage-stage embryos identified numerous nuclear abnormalities within the PCOS groups. Treatment with hyperandrogen led to a significant reduction in blastocyst formation rates. Whereas control embryos predominantly consisted of euploid blastomeres, the majority of cells in the PCOS groups exhibited aneuploidy. Furthermore, differential gene expression was observed in key implantation processes between the control and PCOS groups (Ravisankar et al., 2022; Wang et al., 2023). Nevertheless, the mechanisms underlying the induction of abnormal phenotypes in the offspring of PCOS mouse models due to early-life androgen exposure are not well understood.

Uncertainty remains about potential transcriptional changes in susceptibility genes, especially those from GWAS, in the early embryonic stages of offspring from PNA mice (Wang et al., 2023). This study employed a mouse model of PCOS induced by dihydrotestosterone during late pregnancy, demonstrating that prenatal androgen exposure effectively replicates the clinical characteristics observed in PCOS patients. Transcriptome sequencing was performed on preimplantation embryos derived from PCOS mice undergoing assisted reproductive technology. This study aimed to elucidate the gene expression profiles associated with a prenatal androgen exposure model during the early stages of embryonic development. The findings provide valuable insights into the molecular mechanisms underlying diminished embryo quality and suboptimal reproductive outcomes observed in patients with PCOS. Future research on the mechanisms underlying early embryonic developmental disorders in PCOS may be enhanced by incorporating gene editing techniques, tailored nutritional conditions, and additional protocols informed by the findings of this study.

## Materials and methods

### Animals

All mice in this study were maintained on a C57BL/6J genetic background and sourced from Gempharmatech Co., Ltd (Nanjing, China). The mice were randomly allocated to experimental groups and provided *ad libitum* access to a commercially available pelleted diet (Beijing Keao Xieli Feed Co.) and water. The animals were maintained in controlled environmental conditions of 21°C ± 1°Cwith a humidity of 50% ± 5% under a 12-h light/dark cycle. To induce a prenatally androgenized PCOS-like mice model utilizing DHT, 8-week-old pregnant female mice were subcutaneously administered with DHT at a dosage of 250 µg per animal, once daily, for three consecutive days during E16.5-E18.5. All female offspring served as PCOS-like model mice and control mice. Ethical approval for all animal studies was obtained from the Animal Care and Use Committee of Guangdong Academy of Medical Sciences (S2022-258-01), and the experiments were conducted in accordance with the guidelines set forth by the National Institutes of Health regarding the care and use of animals.

### Metabolic index determination

The body weight of the mouse was recorded weekly. At 8 weeks of age, the mice’s total fat and lean mass were evaluated using the Small Animal Body Composition Analysis and Imaging System (MesoQMR23-060H-I; Nuimag Corp., China), following the manufacturer’s guidelines. The livers and ovaries of mice were isolated and weighed. The liver index was calculated as the percentage ratio of liver weight to body weight.

### Tissue collection

Before dissection, animals underwent an overnight fasting period, generally at 18–20 weeks of age. Mice were anesthetized using isoflurane, and visceral adipose tissues, liver, and ovaries were meticulously excised and promptly weighed using a precision electronic balance. The tissues were then sectioned into appropriately sized portions for snap-freezing, stored at −80°C, fixed in paraformaldehyde for histological examination, or processed according to experimental requirements.

### Blood measurements

The blood samples were subjected to static settlement to achieve complete coagulation and serum precipitation. Following this process, the serum was collected via centrifugation. Serum testosterone levels were quantified using an ELISA kit (Testosterone ELISA Kit, ab108666, Abcam Ltd., UK) in accordance with the manufacturer’s protocol. The assay demonstrated a sensitivity of 0.07 ng/mL, with detection limits ranging from 0.2 ng/mL to 16 ng/mL. The intra-assay coefficient of variation for testosterone was determined to be 5.8%, while the inter-assay coefficient of variation was 10.5%. Insulin concentrations were measured using an ELISA kit (Mouse Ultrasensitive Insulin ELISA Kit, ALPCO, USA), also following the manufacturer’s instructions. This assay exhibited a sensitivity of 0.019 ng/mL, with detection limits between 0.025 ng/mL and 6.9 ng/mL. The intra-assay coefficient of variation for insulin was 3.1%, and the inter-assay coefficient of variation was 5.5%. Serum triglyceride and total cholesterol levels were assessed using enzymatic methods as per the guidelines provided by Applygen Technologies Inc., Beijing, China. The detection range for serum triglycerides was 20 μmol/L to 2 mmol/L, while that for total cholesterol was 20 μmol/L to 5 mmol/L.

### Histopathology and lipid staining

The liver and adipose tissues were preserved in paraformaldehyde (PFA) for 48 h. Subsequently, paraffin-embedded liver sections, with a thickness of 5 μm, were stained using hematoxylin and eosin (H&E) for histopathological analysis. For Oil Red O staining, frozen liver tissues were embedded in an optimal cutting temperature compound. Cryostat sectioning was performed to obtain frozen sections at a thickness of 5 μm, which were then subjected to Oil Red O staining. The Oil Red O working solution was prepared by diluting the stock solution with distilled water in a 3:2 ratio. Sections were incubated in the freshly prepared Oil Red O working solution for 1 hour at room temperature, followed by three washes with distilled water, and subsequently allowed to air dry. Imaging was conducted using a Zeiss Imager M2 equipped with ZEN imaging software. For the quantitative analysis of lipid content, liver sections stained with H&E or Oil Red O were digitized using the Leica-Aperio Versa imaging system. Subsequently, the mean intensity sum and the proportion of the stained area for each sample were quantified employing ImageJ software.

### Assessment of phenotype and estrous cycle

Anogenital distance (AGD) was assessed utilizing a vernier caliper at the time of weaning. Each mouse underwent three measurements, and the mean value was calculated to reduce measurement error. Subsequently, all mice were observed daily until the occurrence of vaginal opening, with the age at vaginal opening recorded in days. After the vaginal opening, monitor the vaginal cytology every day until finish the first whole estrous cycle and record the days of age. Monitoring of the estrous cycle was performed on adult mice aged 6–8 weeks, using vaginal cytology detection over a period exceeding 20 consecutive days (approximately three–four cycles). The stage of the estrous cycle was determined through hematoxylin and eosin staining and microscopy based on vaginal cytology. Two experienced experimenters independently evaluated the cytological results of vaginal smears and statistical analysis was conducted to examine changes in the estrous cycle.

### Ovarian histology and follicle counting

Ovaries were fixed overnight in 4% PFA, followed by dehydration and paraffin embedding. Tissue sections were stained using standard H&E histological protocols. Images were acquired using an inverted microscope. For follicle quantification, ovaries from mice were serially sectioned at a thickness of 5 μm and stained with H&E. Every fifth section of each ovary was examined microscopically. Follicles were categorized as either healthy or atretic based on previously established morphological criteria (Slot et al., 2006). Atretic preantral follicles were identified by a degenerated oocyte accompanied by a disorganized granulosa cell layer with more than 5% apoptotic cells and/or a hypertrophied theca layer. Atretic antral follicles typically contained an intact oocyte, but the granulosa cell layer exhibited more than 5% apoptotic cells, and the theca layer showed hypertrophy. All types of atretic follicles were excluded from the count. Follicle classification followed previously described methods (Myers et al., 2004). Only primordial, primary, secondary, and antral follicles were included in the count when the oocyte was present. Adjacent sections were analyzed to ensure that each follicle, identified by its unique nucleus, was counted only once during the enumeration of larger follicles. The numbers of primordial, primary, secondary, and antral follicles were multiplied by a factor of five to estimate the total follicle count within a single ovary. Additionally, every 10th section of each ovary was examined to ascertain the number of corpora lutea. The adjacent sections were meticulously traced and compared to avoid double-counting the corpora lutea.

### Fertility assay

The fertility assessment in mice commenced at 8 weeks of age and continued for 3 months. Female mice in both the control group (n = 8) and the PCOS group (n = 10) were paired with wild-type fertile male mice of equivalent age, adhering to a mating ratio of 2:1. Daily observations were conducted to detect indications of pregnancy. The time interval, measured in days, from the commencement of the experiment to the birth of the first litter, was recorded. The size of the litter and the sex ratio of the offspring were evaluated.

### Ovarian gonadotropin stimulation and IVF

Three-week-old female PNA mice (n = 6) and control female mice (n = 8) were administered an intraperitoneal injection of 5 IU of pregnant mare serum gonadotropin (PMSG), followed by a subsequent injection of 5 IU of human chorionic gonadotropin (hCG) (Ningbo Sansheng Pharmaceutical Co., Ltd., China) 44 h later. Sixteen hours after the hCG injection, the mice were euthanized. Cumulus-oocyte complexes were extracted from the oviducts, subjected to enzymatic digestion, and treated with hyaluronidase. The number of oocytes was then collected and quantified, with oocyte maturation assessed based on the extrusion of the first polar body (PB1). Male mice were euthanized approximately 14 h after the female mice received the hCG injection, and spermatozoa were collected from the caudal epididymis of two male mice. These spermatozoa were subsequently introduced into c-TYH droplets following a 30-min equilibration period to facilitate sperm activation. Oocytes were then placed in HTF fertilization droplets, with one droplet allocated for each female mouse, and the spermatozoa were subsequently added individually for *in vitro* fertilization. Approximately 6–8 h following the introduction of sperm, the embryos underwent a cleaning process and were subsequently placed into a pre-equilibrated KSOM medium for cultivation. Progression of the fertilized eggs to the two-cell and blastocyst stage was assessed daily, with blastocysts being harvested for subsequent sequencing after 96 h of cultivation. Ten blastocyst-stage embryos from two to three mice were collectively pooled, preserved in TCL buffer containing 1% (v/v) 2-mercaptoethanol, and stored at −80°C for future RNA sequencing analysis.

### RNA-seq library preparation and sequencing

The RNA-seq libraries of blastocyst-stage embryos were generated using the Smart-seq2 protocol for RNA sequencing, as previously described (Ramskold et al., 2012; Conine et al., 2018). Individual embryos were lysed in TCL buffer containing 1% (v/v) 2-mercaptoethanol, followed by RNA isolation utilizing Agencourt RNAClean XP SPRI beads (Beckman Coulter). Complementary DNA (cDNA) was synthesized from full-length polyadenylated mRNAs using Superscript II (Invitrogen), and the signal was amplified through 10 cycles of PCR. Adapters for sample indexing and sequencing were added to the cDNA, which was fragmented by Tn5-mediated tagmentation using the Nextera XT kit (Illumina). The libraries were subsequently purified using magnetic beads. The quality of the cDNA was evaluated with the Agilent2100 BioAnalyzer (Agilent Technologies). Finally, pooled libraries, comprising 96 samples, were sequenced on a NextSeq500 platform (75 cycles, high throughput).

### Analysis of RNA-seq data

This project utilizes the independent filtering software SOAPnuke, which BGI developed for filtering. The process involves the removal of reads containing the adaptor, the elimination of reads with N content exceeding 5%, and the discarding of low-quality reads. The filtered “Clean Reads” are saved in FASTQ format. Processed reads were aligned to mouse reference genome (GRCm38/mm10) by using HISAT, and the expression values were used to remove the possible sample batch effects as described before (Kim et al., 2019). The complete set of RNA-seq raw data has been submitted to the Gene Expression Omnibus (GEO) database and can be accessed under the accession numbers GSE276424.

Genes that exhibited zero counts across all experimental conditions were excluded from the analysis. Principal component analysis (PCA) was conducted using the *R package ggplot2*. Differentially expressed genes (DEGs) were identified through the *R package DESeq2*, which is specifically designed for RNA sequencing analysis. In all differential expression analyses, we applied DESeq2-independent filtering, and P-values were adjusted for multiple hypothesis testing using the Benjamini–Hochberg (BH) false discovery rate (FDR) method. Genes were classified as differentially expressed if the BH-FDR was less than 0.05. Following this, Gene Ontology (GO) functional enrichment and Kyoto Encyclopedia of Genes and Genomes (KEGG) pathway analyses were performed using the DAVID bioinformatics resource (DAVID, https://david.ncifcrf.gov/) to elucidate the functional roles of these DEGs. Additionally, gene set enrichment analysis (GSEA) was conducted using the GSEA software (http://software.broadinstitute.org/gsea/downloads.jsp).

### Statistical analysis

Statistical analyses and graphical representations were conducted utilizing SPSS version 29.0 and GraphPad Prism version 9.5. Prior to conducting analyses, tests for normality were executed. Data from the mouse study are expressed as mean ± standard error of the mean (SEM) for the specified observations. Comparisons between the two groups were assessed using either an unpaired two-tailed Student’s t-test or the Mann-Whitney test. For comparisons across multiple groups, one-way or two-way ANOVA or a mixed-effects analysis model with multiple comparisons test was employed. The respective figure legends provide sample sizes and detailed statistical information for each experiment. Statistical significance was established at a P-value threshold of less than 0.05. Although statistical methods did not predetermine sample sizes, they are consistent with those reported in previous studies. All experiments requiring statistical analysis were performed in triplicate, yielding consistent results.

## Results

### Prenatal androgen exposure causes abnormal lipid metabolic function in mice

**FIGURE 1:**
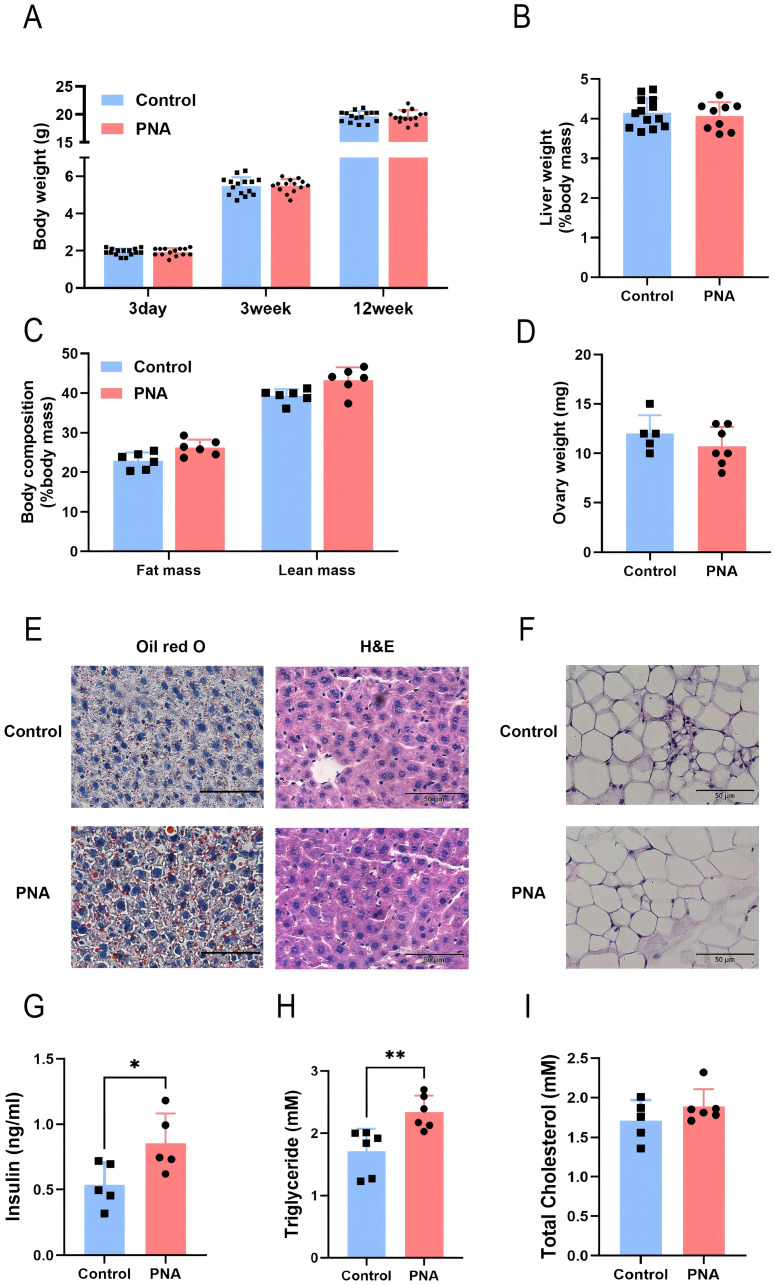
Impact of prenatal hyperandrogen exposure on growth and development in PCOS mice **(A)** Body weights of mice at different weekly ages. n = 15 in Control and n = 14 in PNA **(B)** Liver mass percentage. n = 13 in Control and n = 9 in PNA **(C)** Body composition analysis of mice *in vivo*. n = 6 per group **(D)** Bilateral ovary mass. n = 5 in Control and n = 7 in PNA **(E)** The representative images of Oil red O staining and H&E staining of liver tissue sections. ORO visualizes neutral lipid droplets. n = 5 per group **(F)** Quantitation of liver lipid content determined by ORO staining and shown as percentage of the total histological area **(G)** Average adipocyte diameter calculated from the adipose H&E staining images, n = 5 per group **(H)** Fasting serum insulin level in mice. n = 5 per group **(I)** Fasting serum triglyceride level. n = 6 per group **(J)** Fasting serum total cholesterol levels. n = 6 per group. *P < 0.05, **P < 0.01, and ***P < 0.001.

To investigate the role of intrauterine hyperandrogen exposure in the pathogenesis of polycystic ovary syndrome and to elucidate the specific underlying mechanisms, we developed a mouse model of PCOS characterized by exposure to DHT during late pregnancy. Initially, we assessed the growth and development of the model mice, along with other fundamental indices. The results indicated that, compared to the control group, there were no statistically significant differences in body weight, liver weight, or ovarian weight in the prenatal androgen-exposed group. The body composition analysis indicated a slight increase infat and muscle content, although these changes were not statistically significant (Figures 1A–D). These findings imply that prenatal androgenic exposure did not significantly impact the body mass or the development of various tissues and organs in female mice. Despite the absence of a significant difference in the weight of liver and visceral adipose tissue between PCOS mice and controls, Oil Red O staining revealed an increased accumulation of neutral lipid droplets in the liver tissue of PCOS mice (Figures 1E, F). Additionally, the adipocyte diameter within the visceral adipose tissue of PCOS mice was significantly greater than that observed in the control group (Figure 1G). Furthermore, there was a significant elevation in serum insulin levels, accompanied by a trend towards a mild increase in serum triglyceride and total cholesterol levels (Figures 1H–J). These findings suggest that prenatal hyperandrogenic exposure predominantly affects lipid metabolism within the metabolic system of mice under normal dietary conditions. Prior research has established that adipose tissue serves as the body’s primary organ for lipid storage, with adipocyte size indicating the equilibrium between triglyceride storage and mobilization. When the volume or mass of subcutaneous adipose tissue reaches a critical threshold, lipids may overflow into the liver and other abdominal tissues (de Zegher et al., 2009). The liver, functioning as the body’s metabolic hub, is crucial in maintaining lipid homeostasis and is a key site for metabolic disorders associated with PCOS (Khan et al., 2023; Vidal-Cevallos et al., 2023). As a critical regulatory organ and a reservoir for the synthesis of steroid hormones, abnormal lipid accumulation in hepatic and adipose tissues can lead to elevated levels of steroid hormones, thereby exacerbating other reproductive endocrine disorders in PCOS.

### Prenatal androgen exposure causes abnormal reproductive endocrine function in mice

**FIGURE 2:**
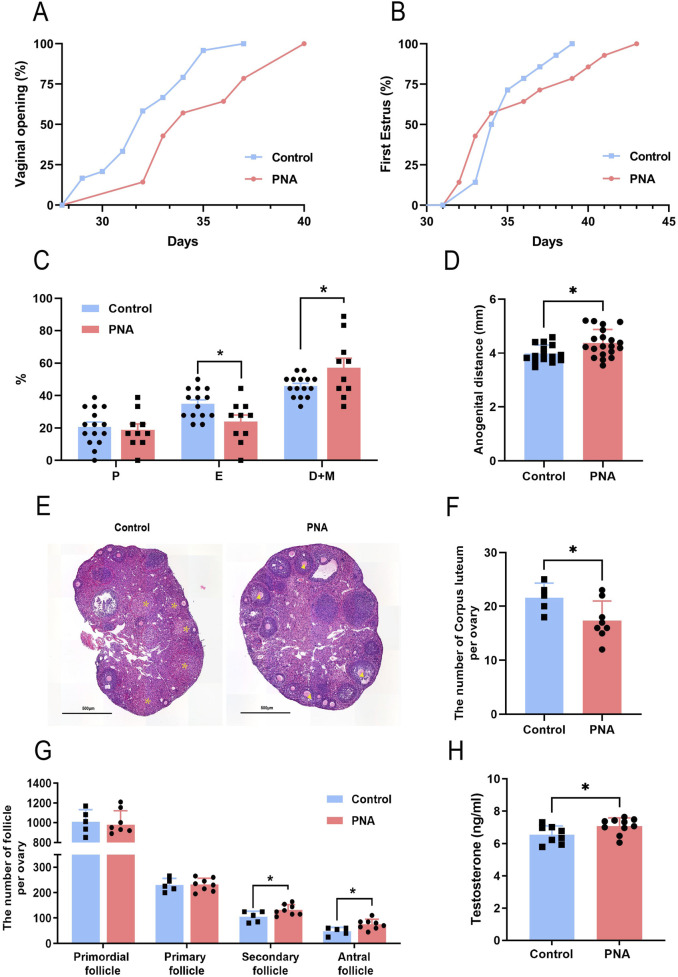
Impact of prenatal hyperandrogen exposure on ovarian function in PCOS mouse models **(A)** Percentage of mice with fully open vaginas. n = 24 in Control and n = 14 in PNA **(B)** Percentage of mice in first estrus. n = 14 per group **(C)** Percentage of mice in each stage of the estrous cycle. n = 15 in Control and n = 10 in PNA **(D)** Distance from vaginal opening to anus in mice. n = 15 in Control and n = 20 in PNA **(E)**The representative images of H&E staining of ovarian tissue. * Labeled corpus luteum, ▲ labeled sinus follicles. n = 5 in Control and n = 6 in PNA **(F)** Number of unilateral ovarian corpus luteum. n = 5 in Control and n = 8 in PNA **(G)** Number of follicles in each stage in the mouse unilateral ovary. n = 5 in Control and n = 8 in PNA **(H)** Serum testosterone levels in mice. n = 8 in Control and n = 10 in PNA. *P < 0.05, **P < 0.01, and ***P < 0.001.

Based on the diagnostic criteria and clinical characteristics of PCOS, we investigated the reproductive phenotype in murine models. The occurrence of vaginal opening and the onset of first estrus were indicative of the progressive development and maturation of the female reproductive system. In comparison to the control group, the onset of vaginal opening was significantly delayed in PCOS mice, with some individuals exhibiting incomplete vaginal opening (Figure 2A). Additionally, the time to first estrus in PCOS mice showed a tendency to be delayed relative to the control group. However, this difference did not reach statistical significance (Figure 2B). Upon examining the estrous cycle of adult female mice, it was observed that PCOS mice exhibited a disrupted estrous cycle, characterized by a shortened estrous phase and an extended inter-estrous/late estrous phase (Figure 2C). The extended vaginal-anal distance suggests that the mice were exposed to a hyperandrogenic environment during the critical period of intrauterine development. Furthermore, the vaginal-anal distance was significantly greater in the PCOS mice compared to the control group (Figure 2D). Through the analysis of serial ovarian sections and follicle counts in the ovarian tissues of both control and PCOS-afflicted mice in adulthood, our findings indicate a slight reduction in the number of corpora lutea in the ovarian tissues of the PCOS group (Figures 2E, F). Conversely, there was a significant increase in the number of secondary and antral follicles (Figure 2G). Additionally, the granulosa cell layer surrounding the antral follicles exhibited a reduced thickness, while the thickness of the membranous mesenchymal stromal cell layer was augmented. Serological analysis in mice revealed a moderate increase in serum testosterone levels within the PCOS group (Figure 2H). These findings suggest the presence of ovarian dysfunction in the PCOS model mice, aligning with certain clinical manifestations observed in PCOS patients.

### Prenatal androgen exposure reduces fertility and causes abnormal embryonic development in mice

**FIGURE 3:**
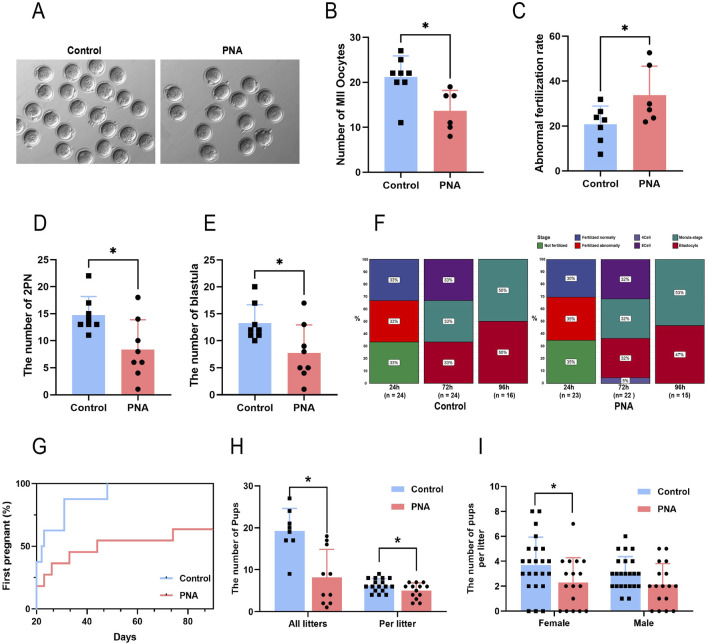
Prenatal exposure to intrauterine hyperandrogenism is linked to reduced fertility **(A)** The images exemplifying the condition of oocytes following superovulation in mice **(B)** Number of mature oocytes. n = 8 per group **(C)** Abnormal fertilization rate of mouse IVF. n = 8 per group **(D)** The number of prokaryotic embryos formed after 24 h of IVF in mice. n = 8 per group **(E)** Number of sacs obtained after 96 h of IVF. n = 8 per group **(F)** Proportion of embryos at developmental stages after different times of *in vitro* culture. n = 8 per group **(G)** The proportion of mice that completed the first litter at different observation times. n = 8 in Control and n = 10 in PNA **(H)** Total number of pups (n = 8 in Control and n = 10 in PNA) and single litter size of mice (n = 17 in Control and n = 13 in PNA) **(I)** Number of offspring of different sexes in a single litter (n = 23 in Control and n = 17 in PNA). *P < 0.05, **P < 0.01, and ***P < 0.001.

Given the observed reduced fertility in clinical patients with polycystic ovary syndrome, we conducted a further investigation into the detrimental effects of intrauterine hyperandrogenic exposure on the fertility of PCOS mouse models, as well as the underlying mechanisms involved. Through the implementation of superovulation and *in vitro* fertilization experiments, we determined that the number of mature metaphase II (MII) oocytes obtained was significantly lower in the PCOS model mice compared to the control group, despite a markedly higher follicle count (Figures 3A, B). Zygotes exhibiting configurations other than 2 PN are typically associated with a heightened risk of chromosomal abnormalities and are generally discarded; thus, we classified these zygotes as abnormally fertilized. The incidence of abnormal fertilization was significantly higher in the PCOS group (Figure 3C). This anomaly led to a significant reduction in the formation of pronuclei and blastocysts (Figures 3D, E), as well as a notable developmental delay in the embryos of the PCOS mice, commencing 48 h post-fertilization (Figure 3F). Previous studies have demonstrated that patients with PCOS exhibit significantly reduced metrics in terms of the number of oocytes retrieved, oocyte maturity, average fertilization rate, and the number of transferable high-quality embryos, compared to patients with normal ovarian infertility (Persson et al., 2019; He et al., 2019). Our findings in model animals corroborated these observations. Subsequently, we permitted free mating between the model and wild-type male mice and evaluated the fertility of both groups by monitoring their litter sizes. The results indicated that mice in the PCOS group exhibited a marked delay in the time to the first offspring (Figure 3G) and a significantly reduced number of litters per litter compared to control mice (Figure 3H). Additionally, the total number of newborn mice within the same period was substantially lower (Figure 3H), with significant reductions observed in both male and female offspring, and no statistically significant difference in the sex ratio (Figure 3I). The results presented indicate that the PCOS model mice, established through prenatal androgen exposure, exhibited characteristics consistent with poor oocyte quality, impaired development embryos, and diminished fertility observed in clinical patients with PCOS. Consequently, the comprehensive utilization of this model holds significant scientific value for investigating the developmental disorders associated with oocytes and embryos in the context of PCOS.

### Prenatal androgen exposure disrupts critical embryonic developmental pathways

**FIGURE 4:**
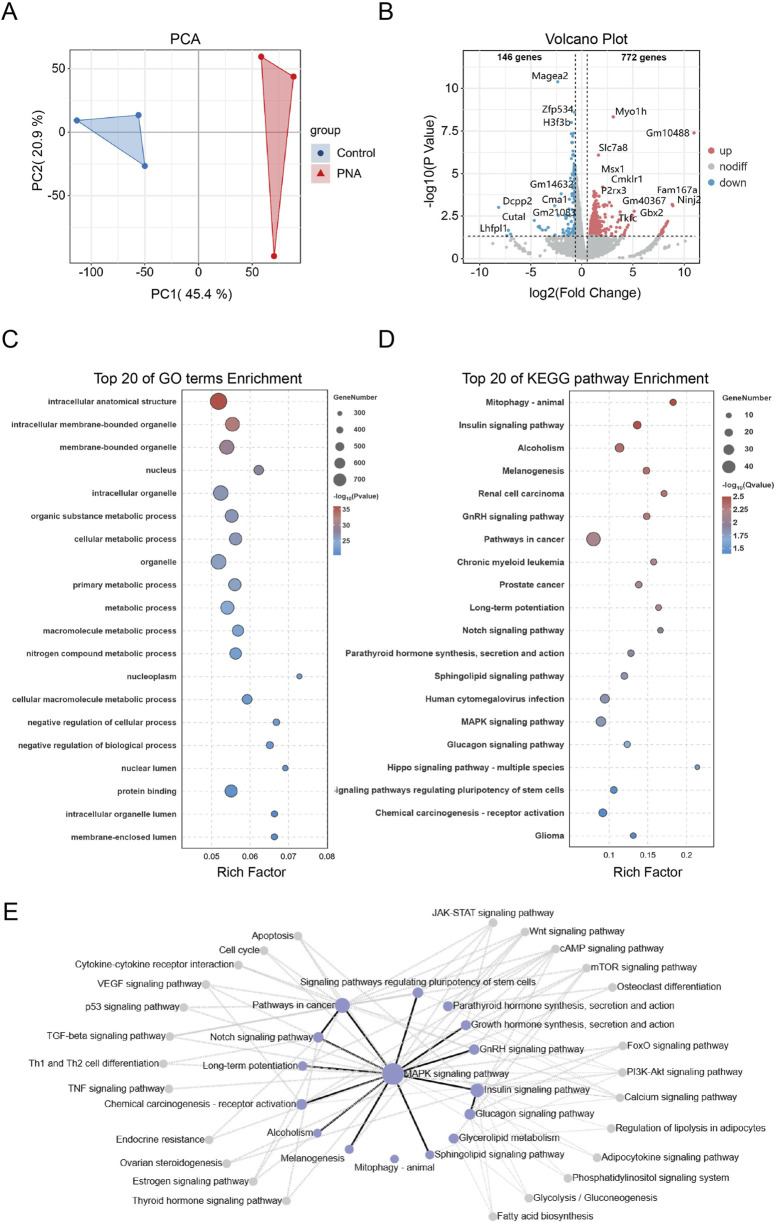
Transcriptomics shows that androgen exposure disrupts embryonic development and energy metabolism **(A)** RNA-seq data from control and PCOS mouse blastocysts demonstrate significant separation **(B)** Volcano plots of differentially expressed genes, with upregulated genes highlighted in red and downregulated genes highlighted in blue **(C, D)** Bubble plots illustrating the top 20 pathways based on significant enrichment of GO and KEGG analyses. The size of each bubble represents the number of genes, while the color indicates the significance of enrichment **(E)** The KEGG pathway interaction network map. Each node indicates a signaling pathway, with purple nodes highlighting the most enriched ones. Lines show interactions: black for positive regulation and gray for negative regulation.

To comprehensively examine the impact of hyperandrogen exposure during late pregnancy on early preimplantation embryonic developmental disorders in a murine model of PCOS, as well as to elucidate the underlying molecular mechanisms, we conducted Smart-SeqII RNA sequencing on blastocyst samples obtained from both control and PCOS groups. Through the application of principal component analysis (PCA) on the RNA-seq data, we identified significant differences between the two groups, with the PC1 and PC2 accounting for 66.3% of the variance (Figure 4A). Differentially expressed genes were defined as P-value <0.05 and absolute value of |*fold change*| > 1.5. The resulting volcano plot revealed 918 differentially expressed genes in the blastocysts of mice with PCOS, comprising 772 upregulated and 146 downregulated genes (Figure 4B). Subsequently, we conducted gene ontology (GO) enrichment analysis on the differentially expressed genes (DEGs), revealing significant enrichment in pathways associated with cytoskeleton synthesis, organelle formation, and other structural components, as well as intracellular energy metabolism (Figure 4C). Additionally, KEGG-based pathway analysis indicated substantial enrichment in pathways related to tissue and organ differentiation and development, glucose and lipid metabolism, and tumorigenesis (Figure 4D). Notably, within the pathway regulatory network, the synthesis pathways of gonadotropin-releasing hormone (GnRH), sex hormones, and other hormones were significantly enriched in mice (Figure 4E). These findings indicate that offspring are exposed to hyperandrogenism during intrauterine development and that gametes retain heritable transcriptional alterations even after being removed from the hyperandrogenic environment postnatally. The presence of genomic transcriptional alterations in gametes and early embryonic tissues of mice with PCOS is closely associated with reproductive endocrine disorders and disruptions in glucose and lipid metabolism observed in adulthood. These findings provide insights into the familial inheritance patterns and offspring susceptibility in PCOS patients. Furthermore, they are crucial for advancing our understanding of the molecular mechanisms underlying reproductive health and their implications in PCOS.

**FIGURE 5:**
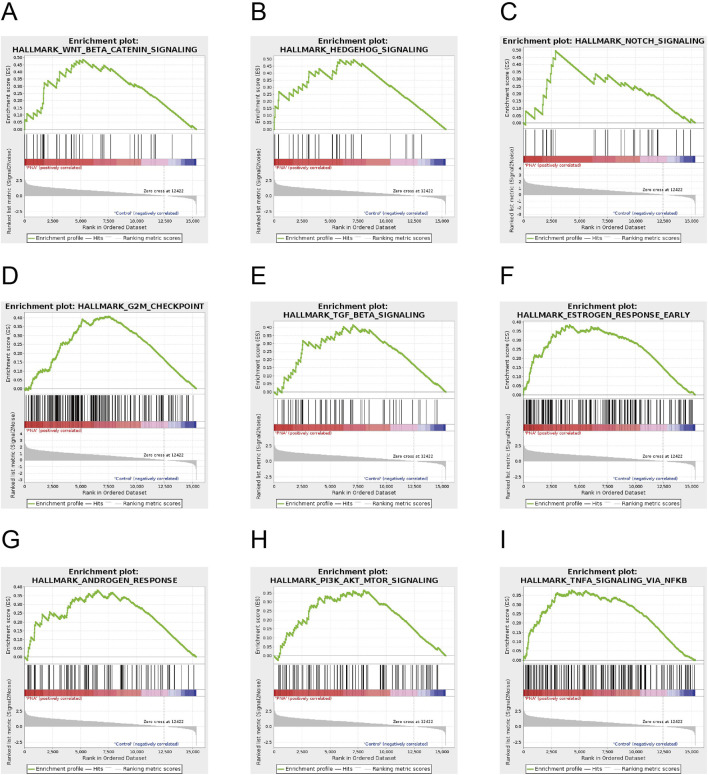
Gene set enrichment analysis of the mouse blastocyst transcriptome **(A–G)** Key pathways in the GSEA-enriched pathway involved in developmental differentiation and steroid hormone secretion, including WNT signaling, HEDGEHOG signaling, and NOTCH signaling, G2M checkpoint, TGF-β signaling, estrogen signaling and androgen signaling pathways **(H, I)** Key pathways in GSEA-enriched pathways involved in cellular energy homeostasis, inflammatory response, including insulin pathway, tumor necrosis factor pathway.

To elucidate the trends in gene set alterations between the two groups of blastocysts, we conducted a gene set enrichment analysis (GSEA). The findings indicated that the expression patterns of the majority of GSEA-enriched pathways were predominantly upregulated. Several pathways intimately associated with developmental differentiation, hormone response, and other aspects of reproductive health exhibited significant activation trends. Specifically, the WNT signaling, HEDGEHOG signaling, NOTCH signaling, G2M checkpoint, TGF-βsignaling, estrogen signaling, and androgen signaling pathways all demonstrated notable upregulation trends (Figures 5A–G). These alterations indicate a favorable adaptation of gametes in PCOS-afflicted mice to early-life adverse conditions. Furthermore, these adaptations may elucidate a potential mechanism underlying the observed decline in oocyte and embryo quality, as well as the heightened long-term risk of metabolic disorders, including obesity, in patients with PCOS. Furthermore, key pathways implicated in cellular energy homeostasis and inflammatory responses, including the insulin signaling pathway and the tumor necrosis factor (TNF) pathway, exhibited a trend of upregulation (Figures 5H, I). This observation substantiates the hypothesis that insulin resistance and a chronic inflammatory state contribute to the pathogenesis of polycystic ovary syndrome. The transcriptome profiling of blastocysts from PCOS mice indicated that early life exposure to hyperandrogenism results in significant transcriptional alterations in embryo genomes. These alterations predominantly affect pathways related to energy metabolism, tissue differentiation, and development, and are closely linked to the manifestation of adverse reproductive and metabolic phenotypes in PCOS.

## Discussion

In this study, we demonstrated that prenatal exposure to elevated androgen levels during late gestation results in ovarian dysfunction and metabolic disorders in later life, closely mirroring the characteristics of PCOS. Additionally, individuals with PCOS exhibit significantly diminished metrics regarding the number of oocytes retrieved, oocyte maturity, average fertilization rate, and the number of transferable high-quality embryos, in comparison to individuals with normal ovarian infertility (Persson et al., 2019; He et al., 2019). Our findings corroborate that the prenatal androgen-exposed mouse model accurately replicates these outcomes, particularly the reduced quality of oocytes and embryos, as well as impaired early embryonic development following ovulation induction and *in vitro* fertilization procedures. Through transcriptomic analysis of blastocysts in a murine model of PCOS, we have elucidated the gene expression profile associated with prenatal androgen exposure in blastocysts. These findings confirm the intergenerational inheritance of polycystic ovary syndrome in mice induced by prenatal androgen exposure, and also provide theoretical support for the theory that PCOS is a disease of embryonic origin. Alterations in the fundamental signaling pathways integral to development, as induced by prenatal androgen exposure, can be imprinted in the oocytes and transmitted to early embryonic tissues. These abnormalities may contribute to reproductive dysfunction and the development of PCOS in female offspring. In our study, we observed that significant alterations in the early embryo were predominantly associated with the hormone synthesis pathway, Insulin signaling pathway, and immune-related pathway, and stem cell differentiation pathway. Based on the potential pathogenesis-related pathways of PCOS identified through transcriptomic analyses, we have reviewed previous studies that involve these pathways. We selected specific genes and pathways for focused discussion, drawing upon perspectives from published literature.

It is worth noting that the gonadotropin-releasing hormone biosynthetic and response pathways are significantly activated in mouse embryos with PCOS. GnRH serves as the primary regulator of ovarian function and is influenced by a variety of intrinsic and extrinsic factors. Consequently, any disturbances in these regulatory mechanisms can result in reproductive endocrine disorders (Chaudhari et al., 2018). Previous studies have shown that women with PCOS consistently exhibit an increased frequency of luteinizing hormone release, driven by abnormal GnRH secretions. This irregular pattern of GnRH release exacerbates hyperandrogenemia and contributes to the disrupted reproductive cycles that are characteristic of PCOS(37). Studies conducted on animals have likewise demonstrated that PNA leads to modifications in the presynaptic organization of the GABAergic network connected to GnRH neurons, as well as alterations in the postsynaptic response of GnRH neurons. These changes contribute to reproductive dysfunction in adulthood of PCOS model (Berg et al., 2018; Zhou et al., 2025). GNAQ modulated the expression of its downstream gene within hypothalamic neurons and directly influenced the expression and secretion of GnRH via the calcium and protein kinase C signaling pathways (Zhu et al., 2022) and kisspeptin-GPR54 signaling pathway (Babwah et al., 2015). The observed increase in AgRP neurons within the arcuate nucleus of PNA sheep and rodents suggests a potential mechanism by which testosterone may directly impact energy balance (Sheppard et al., 2011; Watanabe et al., 2023). In our study, *Agrp* and *Gnaq* were identified as significantly differentially expressed genes in the blastocysts of PNA mice, indicating the potential for developmental hypothalamic programming to induce alterations in the secretory function of hypothalamic neurons in PNA mice during adulthood.

The other finding is worth noting that the significant enrichment of various insulin signaling pathways in PNA embryos. This finding is consistent with previous studies suggesting that PCOS patients experience chronic insulin resistance over the long term, characterized by increased hyperinsulinemia and reduced sensitivity of insulin receptors (Guo et al., 2023). A post-binding defect in receptor signaling differentially impacts metabolism in traditional insulin target tissues as well as the ovary. Insulin acts as a co-gonadotropin via its specific receptor to regulate ovarian steroidogenesis, and insulin signaling in the brain has been shown to play a crucial role in ovulation and the regulation of body weight (Diamanti-Kandarakis and Dunaif, 2012). Studies indicate that insulin’s mitogenic signaling may influence steroid production and glucose balance. In PCOS patients, higher *HRAS* and *KRAS* expression in subcutaneous fat is linked to increased testosterone levels and lower metabolic indicators like BMI, fasting glucose, insulin, and HOMA-IR (Xu et al., 2015). A genome-wide association study (GWAS) examining 636,797 autosomal single nucleotide polymorphisms (SNPs) from a cohort of 1,221 individuals identified prominent pathways and gene sets linked to PCOS, notably including the *GNAQ* gene. Diplotypes within the promoter region of *GNAQ* have been found to be associated with insulin resistance and obesity in individuals with PCOS (Shim et al., 2015; Klenke et al., 2010). In our study, *Hras*, *Kras*, and *Gnaq* were identified as enriched differentially expressed genes in the blastocysts of PNA mice, suggesting a potential for developmental abnormalities in critical organs responsible for maintaining glucose and lipid metabolic homeostasis in adulthood.

In the current study, transcriptome differential enrichment analysis identified a notable enrichment of various immune-related pathways, such as TNF and IL6 signaling, alongside metabolic process pathways, including lipolytic and adipogenic gene expression. These observations align with previous research indicating that patients with PCOS experience chronic low-grade inflammation over an extended duration, accompanied by disruptions in glucose and lipid metabolic homeostasis. Immune-related pathways play a critical role in ovarian ovulation; any abnormal increase or infiltration of immune cells may adversely affect follicular development, potentially resulting in ovulatory disorders (Rostamtabar et al., 2021). Tumor necrosis factor and interleukins have been observed both in the circulating blood and localized within the ovarian tissues of PCOS patients (Adams et al., 2016; Escobar-Morreale et al., 2005). These significant inflammatory factors have additionally been identified to modulate lipid metabolism-related genes, including the lipolytic gene *PRKACA* (Klenke et al., 2010). The enrichment of IL6, TNFa, and other immune pathways in PNA embryos suggest that the dysregulated expression of inflammatory pathways may not only lead to localized ovarian dysfunction but also potentially convey the risk of PCOS to the offsprings through the oocyte.

The complexities associated with sample collection and the limitations of current sequencing technologies have resulted in a paucity of research on early embryos in animal models of PCOS. Consequently, numerous critical inquiries regarding the alterations occurring during the embryonic period of PCOS remain difficult to address within the scope of this study. Given that a substantial proportion of patients with PCOS achieve successful conception through assisted reproductive technologies, we opted to exclude developmentally arrested PCOS embryos from transcriptional sequencing analysis. Although this decision might have omitted transcriptional variants associated with severe developmental abnormalities, it ensures that the data more accurately reflect the typical condition of PCOS embryos. Within our sample collection, which predominantly progresses to the blastocyst stage, we still observed numerous expression changes in pathways related to impaired trophoblast differentiation. *Esrrb* and *Elf5* are crucial in maintaining trophoblast stem cells, essential for differentiating into various cell lineages that support placental function. Deleting either *Elf5* or *Esrrb* results in embryonic lethality during early to mid-gestation in mice. Additionally, placentas from PCOS mouse models and PCOS patients exhibit a significantly reduced number of trophoblast precursors (Lu et al., 2024). The upregulation of genes such as Esrrb and Elf5 in PNA embryos indicates an increased risk of impaired trophoblast differentiation and miscarriage in the PCOS models examined in our study.

## Conclusion

In summary, the current study establishes that prenatal intrauterine hyperandrogenic exposure is an effective approach for creating a model of polycystic ovary syndrome that faithfully replicates its hallmark characteristics, including oocyte developmental disorders, compromised embryo quality, and decreased fertility. Furthermore, transcriptomic analysis of mouse blastocysts has provided insights into the abnormalities in hormone synthesis, metabolic processes, and inflammatory pathways, which were documented and transmitted to the offspring’s early embryos. The examination of early embryos in a PCOS mouse model is crucial for a comprehensive understanding of the pathogenesis of this condition, as it contributes to our knowledge of the disease and facilitates the development of more effective prevention and management strategies.

## Data Availability

The datasets presented in this study can be found in online repositories. The names of the repository/repositories and accession number(s) can be found in the article/supplementary material.
